# Breakthrough invasive aspergillosis and diagnostic accuracy of serum galactomannan enzyme immune assay during acute myeloid leukemia induction chemotherapy with posaconazole prophylaxis

**DOI:** 10.18632/oncotarget.25477

**Published:** 2018-06-01

**Authors:** Claire Calmettes, Frederic Gabriel, Elodie Blanchard, Vincent Servant, Stéphane Bouchet, Nathanael Kabore, Edouard Forcade, Camille Leroyer, Audrey Bidet, Valérie Latrabe, Thibaut Leguay, Stephane Vigouroux, Reza Tabrizi, Dominique Breilh, Isabelle Accoceberry, Manuel Tunon de Lara, Arnaud Pigneux, Noel Milpied, Pierre-Yves Dumas

**Affiliations:** ^1^ Department of Hematology and Cell Therapy, University Hospital, F-33000 Bordeaux, France; ^2^ Laboratory of Mycology, University Hospital, F-33000 Bordeaux, France; ^3^ Department of Respiratory Diseases, University Hospital, F-33000 Bordeaux, France; ^4^ Pharmacy, University Hospital, F-33000 Bordeaux, France; ^5^ Department of Clinical Pharmacology, University Hospital, F-33000 Bordeaux, France; ^6^ Medical Information Department, University Hospital, F-33000 Bordeaux, France; ^7^ Department of Infection Control, University Hospital, F-33000 Bordeaux, France; ^8^ Laboratory of Hematology, University Hospital, F-33000 Bordeaux, France; ^9^ Thoracic and Cardiovascular Imaging Department, University Hospital, F-33000 Bordeaux, France

**Keywords:** acute myeloid leukemia, posaconazole, invasive aspergillosis, galactomannan enzyme immunoassay, sensitivity

## Abstract

Posaconazole prophylaxis has demonstrated efficacy in the prevention of invasive aspergillosis during prolonged neutropenia following acute myeloid leukemia induction chemotherapy. Antifungal treatment decreases serum galactomannan enzyme immunoassay diagnostic accuracy that could delay the diagnosis and treatment.

We retrospectively studied patients with acute myeloid leukemia who underwent intensive chemotherapy and antifungal prophylaxis by posaconazole oral suspension. Clinical, radiological, microbiological features and treatment response of patients with invasive aspergillosis that occurred despite posaconazole prophylaxis were analyzed. Diagnostic accuracy of serum galactomannan assay according to posaconazole plasma concentrations has been performed.

A total of 288 patients with acute myeloid leukemia, treated by induction chemotherapy, who received posaconazole prophylaxis for more than five days were included in the present study. The incidence of invasive aspergillosis was 8% with 12 (4.2%), 8 (2.8%) and 3 (1%), possible, probable and proven invasive aspergillosis, respectively. Posaconazole plasma concentration was available for 258 patients. Median duration of posaconazole treatment was 17 days, and median posaconazole plasma concentration was 0.5 mg/L. None of patients with invasive aspergillosis and posaconazole concentration ≥ 0.5 mg/L had a serum galactomannan positive test. Sensitivity of serum galactomannan assay to detect probable and proven invasive aspergillosis was 81.8%. Decreasing the cut-off value for serum galactomannan optical density index from 0.5 to 0.3 increased sensitivity to 90.9%.

In a homogenous cohort of acute myeloid leukemia patients during induction chemotherapy, increasing the posaconazole concentration decreases the sensitivity of serum galactomannan assay.

## INTRODUCTION

Treatment of acute myeloid leukemia (AML) is generally divided in 2 steps: induction and consolidation. This treatment has not been really modified for 40 years and most of the prognostic improvement came from supportive care and allogeneic stem cell transplant [1]. The standard regimen of AML induction chemotherapy is a combination of an anthracycline with continuous infusion of cytarabine. This treatment leads to a deep and prolonged neutropenia which is a classical risk factor of invasive fungal disease (IFD), mainly invasive aspergillosis (IA).

Establishing early diagnosis of IA is a major issue hampered by poor clinical signs and the difficulty of mycological documentation. The diagnosis is based on a set of arguments: host factor, clinical and radiological patterns and mycological criteria for assignment of possible, probable and proven IA, according to European Organization for Research and Treatment of Cancer/Mycoses Study Group (EORTC/MSG) criteria [2].

Direct microbiological diagnosis is rare in immunocompromised patients, requiring the use of indirect test such as serum Galactomannan (GM) enzyme immunoassay whose optical density index (GM-ODI) values are correlated with fungal burden and *Aspergillus*-related disease progression [3]. Relevance of this test in IA diagnosis is criticized but remained the corner stone of decision tree in the management of fever in neutropenic patient, particularly during AML induction chemotherapy before prophylaxis area. This relevance is questionable since 2006 and the use of posaconazole as antifungal prophylaxis to prevent IFD during the neutropenic period following AML induction chemotherapy.

The current study aims to describe the breakthrough IA despite antifungal prophylaxis by posaconazole in a large cohort of AML patients during the induction chemotherapy. Moreover, this homogeneous population allowed us to determine the diagnostic accuracy of serum GM-ODI according to posaconazole plasma concentration in this specific context.

## RESULTS

### Characteristics of patients and treatments

Five hundred and eighty patients were initially identified for evaluation. We included 288 patients according to flow chart (Supplementary Figure 1) with a median follow-up about 13 months (range 1;75). Demographic and treatment characteristics of these patients are shown in Table 1. Briefly, median age was 62 years, 4.1% of non-IA and 17.4% of IA patients had a history of chronic respiratory disease. All patients received standard dose of cytarabine, with daunorubicin for 35.1% or idarubicin for 64.6% of patients, 4.5% of patients received high dose cytarabine as second course of treatment for primary induction failure. Regimens of first and second induction courses are detailed in Supplementary Table 1. Median duration of neutropenia < 1×10^9^/L and < 0.5×10^9^/L was respectively 24 and 21 days for non-IA patients and 31.5 and 29 days for IA patients. Deep neutropenia with absolute neutrophil count lower than 0.1×10^9^/L for at least 1 day occurred in 38.9% of non-IA patients and 47.8% of IA patients. Two-hundred thirty-seven patients (83.8%) received granulocyte-colony-stimulating-factor during induction.

**Table 1:** Patients characteristics

	Total	Absence of IA-n (%)	IA-n (%)
	N=288	265 (92%)	23 (8%)
**Age (years)**			
Median (IQR)	62 (50-67.5)	62 (51.5-68.5)	56 (49.5-68)
Range	17;83	17;83	22;73
**Male gender-n (%)**	165 (57.3)	149 (56.2)	16 (69.6)
**Performance *status*-n (%)**			
0-1	219 (76)	204 (77)	15 (65.2)
≥ 2	59 (20.5)	51 (19.2)	8 (34.8)
Missing data	10 (3.5)	10 (3.8)	0
**Comorbidities-n (%)**			
Chronic respiratory disease	15 (5.2)	11 (4.1)	4 (17.4)
Serum creatinine > 1.3 mg/dL	1 (0.3)	1 (0.4)	0
Cirrhosis	0	0	0
*Diabetes mellitus*	18 (6.3)	16 (6)	2 (8.7)
Host factor except neutropenia	0	0	0
**WBC (G/L) at diagnosis**			
Median (IQR)	5 (2.1-33.1)	5 (2.1-32.2)	4.1 (2-50.6)
Range	0.3;269.2	0.3;269.2	0.4;134
**ANC (G/L) at diagnosis**			
Median (IQR)	1.1 (0.4-3.7)	1.16 (0.4-3.7)	0.9 (0.5-3.2)
Range	0;81.6	0;81.6	0;12
**FAB classification-n (%)**			
0-2	92 (31.9)	86 (32.5)	6 (26.1)
3	19 (6.6)	19 (7.2)	0
4-5	68 (23.6)	62 (23.4)	6 (26.1)
6-7	24 (8.4)	20 (7.5)	4 (17.4)
No FAB classification	85 (29.5)	78 (29.4)	7 (30.4)
**WHO subtype-n (%)**			
*De novo*	230 (79.9)	213 (80.4)	17 (73.9)
Secondary	58 (20.1)	52 (19.6)	6 (26.1)
MDS-related	14 (4.9)	12 (4.5)	2 (8.7)
MPN-related	20 (6.9)	19 (7.2)	1 (4.3)
tAML	24 (8.3)	21 (7.9)	3 (13)
**Cytogenetics-n (%)**			
Favorable	49 (17)	46 (17.3)	3 (13)
Intermediate	180 (62.5)	166 (62.6)	14 (60.8)
Adverse	59 (20.5)	53 (20)	6 (26)
**Induction chemotherapy-n (%)**			
Daunorubicin-based	101 (35.1)	90 (34)	11 (47.8)
Idarubicin-based	186 (64.6)	175 (66)	11 (47.8)
Other	1 (0.3)	0	1 (4.4)
**Reinduction chemotherapy-n (%)**			
High-dose cytarabine	13 (4.5)	8 (3)	5 (21.7)
**Duration of ANC < 1×10^9^/L (days)**			
Median (IQR)	24 (20.5-29.5)	24 (20.5-28.5)	31.5 (22-43)
Range	8;59	8;52	11;59
**Duration of ANC < 0.5×10^9^/L (days)**			
Median (IQR)	21 (18.5-27)	21 (17.5-27)	29 (23.5-40)
Range	3;51	3;49	10;51
**ANC < 0.1×10^9^/L**			
n (%)	117 (40.6)	103 (38.9)	11 (47.8)
Median duration (IQR) (days)	8.5 (3-14.5)	8 (3-14.5)	11 (7-23.5)
Range	1;29	1;29	2;24
**Grade 4 mucositis-n (%)**	28 (9.7)	23 (8.7)	5 (21.7)
**Response to treatment-n (%)**			
CR-CRi after first induction course	223 (77.4)	220 (83)	16 (69.5)
CR-CRi after second induction course	4 (1.4)	4 (1.51)	0

IA: invasive Aspergillosis, WBC: white blood cell count, ANC: absolute neutrophil count, WHO: World Health Organization, MDS: myelodysplastic syndrome, MPN, myeloproliferative neoplasm, tAML: therapy-related acute myeloid leukemia, FAB: French-American-British classification, IQR: Interquartile range, CR: complete remission, CRi: complete response with incomplete blood recovery.

### Antifungal prophylaxis

All patients received posaconazole as prophylaxis treatment, with a median duration of treatment of 17 days (IQR 12.5-22, range 5;64). Posaconazole prophylaxis has been discontinued in 8 (2.8%) patients for adverse events including 6 liver toxicities and in 124 (43%) patients for empirical or curative antifungal treatment. Two hundred and fifty-eight patients had at least one posaconazole plasma dosage with a mean of 2.7 dosages by patient (range 1-9). We analyzed 701 posaconazole plasma concentrations. Considering the highest individual plasma concentration, median of posaconazole plasma concentration was 0.5 mg/L (IQR 0.3-0.8, range 0.1;2.4) for the whole cohort but 0.46 mg/L, 0.32 mg/L and 0.18 mg/L in possible, probable and proven IA patients, respectively.

### Characteristics and factors associated with IA

The overall incidence of possible, probable and proven IA was 8% (Table 2). Twelve patients had possible IA, characterized by clinical criteria without mycological or histological criteria. Among the 8 patients with probable IA, serum GM-ODI was ≥ 0.5 in 7 patients (87.5%) and GM-ODI in bronchoalveolar lavage (BAL) was ≥ 1 in 4 patients among five patients tested. For the 3 patients with proven IA, the diagnosis of IA was made by lobectomy or bronchial biopsy. Among them, 2 had serum GM-ODI ≥ 0.5 and 2 had GM-ODI ≥ 1 in BAL. Clinical, mycological and histological criteria used for EORTC/MSG 2008 sub group assignment are described in Table 2. Median delay from the beginning of chemotherapy to diagnosis of IA was 26 days (IQR 21-52, range 7;77). Median duration of absolute neutrophil count < 0.5×10^9^/L was 25 days, 36 days and 44 days for possible, probable and proven IA, respectively. Clinical presentations were poor, but fever was constant, with inconstant cough, chest pain or dyspnea. Half of the patients were still febrile after 72 hours of antibacterial therapy and the other half became febrile after 48h of apyrexia under antibacterial therapy. Computerized tomography (CT) scans were performed one or two days after the onset of symptoms. The most frequent CT signs were nodules with or without halo, ground glass opacities and alveolar consolidation. In univariate analysis, factors significantly associated with these 23 possible, probable and proven IA are described in Table 3. In multivariate analysis, factors associated with IA were previous history of chronic respiratory disease (OR 5.79, ^95%^CI [1.51-22.24], p=0.0105) and duration of absolute neutrophil count < 0.5×10^9^/L longer than 21 days (OR 4.34, ^95%^CI [1.39-13.57], p=0.0118) whereas patients with a posaconazole plasma concentration > 0.5 mg/L had a lower risk of IA (OR 0.22, ^95%^CI [0.07-0.67], p=0.0082) (Table 3). Treatments of IA are shown in Table 4. Twenty-two among the 23 IA patients received antifungal treatment with voriconazole whereas one exclusively received liposomal amphotericin B treatment. The 3-month and one-year mortality rates were respectively 18% and 73% for the probable and proven IA, versus 5.3% and 18.1% in the non-IA population. Six patients died during the month following AML chemotherapy, two from a non-IA infection, 3 from AML progression, and one from cerebral hemorrhage.

**Table 2:** Invasive aspergillosis characteristics

		Possible IA-n (%)	Probable IA-n (%)	Proven IA-n (%)
		12 (4.2)	8 (2.8)	3 (1)
**AML**	**Duration of ANC < 1×10^9^/L**			
	Median (IQR)	28 (22-34)	39 (32-48)	49 (21-55)
	Range	11;47	22;59	21;55
	**Duration of ANC < 0.5×10^9^/L**			
	Median (IQR)	25 (21-30)	36 (28.5-41)	44 (21-51)
	Range	10;40	21;48	21;51
	**AML treatment response-n (%)**			
	CR-CRi	10 (83.3)	4 (50)	2 (66.7)
	Primary or secondary induction failure	1 (8.3)	4 (50)	1 (33.3)
	Death before evaluation	1 (8.3)	0	0
**Prophylaxis**	**Posaconazole duration (days)**			
	Median (IQR)	13 (9-26)	11 (8-17.5)	15 (7-20)
	Range	5;64	5;41	7;20
	**Posaconazole plasma concentration (mg/L)^a^**			
	Median (IQR)	0.46 (0.4-0.65)	0.32 (0.2-0.37)	0.18 (0.1-0.4)
	Range	0.3;1	0.22;2.4	0.1;0.4
**Clinical criteria**	**Clinical signs**			
	Fever	12 (100)	8 (100)	3 (100)
	Cough	3 (25)	2 (25)	1 (33.3)
	Chest pain	3 (25)	0	0
	Dyspnea/crackles	4 (33.3)	3 (37.5)	0
	**Chest CT signs**			
	Nodule with halo sign	6 (50)	5 (62.5)	2 (66.7)
	Nodule without halo	9 (75)	3 (37.5)	1 (33.3)
	Cavity / Air crescent sign	0	0	0
	Ground glass opacities	5 (41.7)	3 (37.5)	2 (66.7)
	Alveolar consolidation	6 (50)	1 (12.5)	1 (33.3)
	Centrilobular micronodules	3 (25)	2 (25)	0
	**Bronchoscopy**			
	Performed/indicated-n	10/12	6/8	3/3
	Normal	5 (50)	4 (66.7)	1 (33.3)
	Inflammation	5 (50)	2 (33.3)	2 (66.7)
	Tracheobronchitis^b^	1(10)	0	2 (66.7)
**Mycological criteria**	**Serum GM-ODI ^c^**			
	> 0.5-n (%)	0	7 (87.5)	2 (66.7)
	Mean (Range)	0.23 (0.11;0.46)	1.7 (0.23;5.2)	3.0 (0.37;7.6)
	**GM-ODI in BAL**			
	Performed/bronchoscopy-n	8/10	5/6	2/3
	GM-ODI > 1-n (%)	0	4 (80)	2 (100)
	**Mycological test in BAL**			
	Performed/bronchoscopy-n	10/10	6/6	3/3
	Positive direct examination	0	0	1 (33.3)
	Positive culture	0	1 (16.6)	3 (100)
	Identification	none	1 *A. fumigatus*	2 *A. fumigatus*
				1 *A. niger*
**Histological criteria**	**Examination of sterile material**			
	Lobectomy or bronchial biopsy	0	0	IFD

IA: invasive Aspergillosis, AML: acute myeloid leukemia, IQR: Interquartile range, CR: complete remission, CRi: complete response with incomplete blood recovery, CT: computerized tomography, BAL: bronchoalveolar lavage fluid, GM-ODI: Galactomannan optical density index, IFD: invasive fungal disease, ANC: absolute neutrophil count.

^a^ Highest posaconazole plasma concentration obtained by patient before diagnosis has been considered.

^b^ Ulceration, nodule, pseudo membrane, eschar.

^c^ Highest serum GM-ODI value obtained by patient before diagnosis of IA has been considered.

**Table 3:** Univariate and multivariate analyses of factors associated with possible, probable and proven IA under posaconazole prophylaxis

	Univariate analysis			Multivariate analysis		
	OR	^95%^CI	p	OR	^95%^CI	p
Age (years) ≥ 62	0.59	0.24-1.40	0.232	-	-	-
Deep neutropenia (ANC < 0.1×10^9^/L)	1.38	0.58-3.25	0.465	-	-	-
Chronic respiratory disease	4.86	1.26-15.81	0.024	5.79	1.51-22.24	0.0105
Grade IV mucositis	2.92	0.90-8.13	0.072	-	-	-
Posaconazole plasma concentration ≥ 0.5 mg/L ^a^	0.22	0.06-0.60	0.002	0.22	0.07-0.67	0.0082
Duration of ANC < 0.5×10^9^/L > 21 days	4.37	1.59-15.38	0.003	4.34	1.39-13.57	0.0118

^a^ Highest posaconazole plasma concentration obtained by patient before diagnosis has been considered, ANC: absolute neutrophil count, OR: Odds ratio, CI: confidence interval.

**Table 4:** Treatment and evolution of IA patients

	Possible IA	Probable and proven IA
	n=12	n=11
**Antifungal treatment-n ^a^**		
Liposomal amphotericin B	6	3
Caspofungin	3	1
Voriconazole	11	10
**Duration of treatment (days)-Mean (range)**		
Liposomal amphotericin B	7 (1;12)	8 (2;20)
Caspofungin	6 (3;9)	11 (3;25)
Voriconazole	100 (2;235)	130 (42;261)
**Causes of Voriconazole discontinuation-n ^b^**		
Complete response	10	5
Failure	0	1
Death	1	4
**Clinical Response (days)-Mean (range)**		
Time to apyrexia	6 (1;14)	11 (1;19)
**TDM evaluation-n**	7	9
Aggravation	0	2

^a^ Each patient can receive several treatments, ^b^ One patient with possible IA did not receive voriconazole, but only liposomal amphotericin B, IA: invasive aspergillosis.

### Diagnostic accuracy of the serum GM assay

We then analyzed the diagnostic accuracy of serum GM assay for breakthrough IA in our population. Sensitivity and specificity of serum GM test were calculated considering the results of 2297 tests performed on samples obtained from 288 patients. Sensitivity was defined by the proportion of patient with IA diagnosed by mycological criteria other than serum GM-ODI (*i.e*. positive culture or GM-ODI in BAL). Sensitivity of serum GM assay for the detection of probable and proven IA was 81.8% ^95%^CI [48.2-97.7] and specificity was 96.4% ^95%^CI [93.5-98.3].

Ten patients were identified as false positives with a median follow-up about 31 months (range 2;62). Median age was 63.5 years, 40% of male gender, 80% has an ECOG < 2 at diagnosis, and one had an history of diabetes mellitus. Median duration of absolute neutrophil count < 1×10^9^/L or < 0.5×10^9^/L were both 22 days. Deep neutropenia with absolute neutrophil count lower than 0.1×10^9^/L for at least 1 day occurred in 50% of these ten patients. Median duration of posaconazole prophylaxis was 18 days (range 7;27) and median of posaconazole plasma concentration was 1 mg/L (range 0.3;1.31). Among these ten patients, two (20%) developed a fungemia (with *Candida glabrata* or *Geotrichum capitatum*) during the neutropenic period following the induction chemotherapy. We did not find well-known risk factors of false positive GM assay such as piperacillin-tazobactam, high-dose immunoglobulin or parenteral nutrition.

To describe the effect of posaconazole on diagnostic accuracy of serum GM assay to detect IA, we considered the highest posaconazole plasma concentration obtained by patient before possible, probable or proven IA diagnosis. Nine among the 124 patients (7.2%) with posaconazole concentration < 0.5 mg/L developed an IA: 6 were probable or proven with 5 serum GM positive test (ODI: 5.2, 0.54, 0.98, 1.56, 7.61) and 1 serum GM negative test (ODI: 0.37). Among the 134 patients with posaconazole plasma concentration ≥ 0.5 mg/L, 4 developed an IA (2.9%) but none of them had a serum GM positive test. Taken together, these results suggest that the diagnostic accuracy of serum GM assay is impaired by high posaconazole concentration.

### Variation of serum GM-ODI cut-off positivity

**Figure 1:**
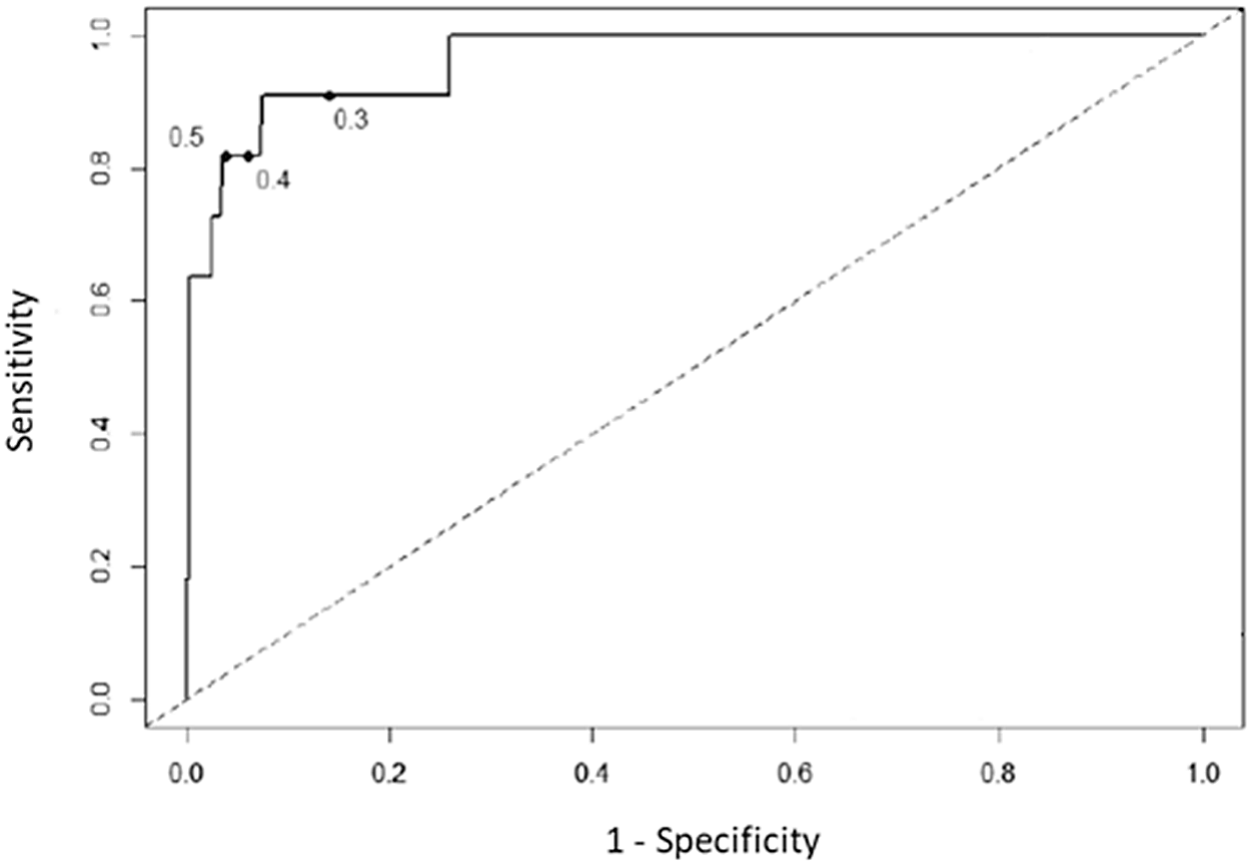
Receiver operator characteristic curves, using multiple index cutoff values to define positivity The index cutoff value defining positivity decreases from the highest to lowest value as the curve moves from left to right.

In view of this decreased diagnostic accuracy of the serum GM assay during effective posaconazole prophylaxis, we wondered whether decreasing the index cutoff value (ICV) could increase the sensitivity of serum GM test during posaconazole prophylaxis. Receiver operator characteristic curves analysis showed that serum GM assay accuracy was still effective to discriminate probable and proven IA (area under the curves =0.963). We showed an increase of sensitivity from 81.8%, ^95%^CI [48.2-97.7] to 90.9%, ^95%^CI [58.7-99.8] when ICV decreased from 0.5 to 0.3 (Figure 1). With this new ICV, the serum GM test became positive for 4 additional patients who had serum GM negative test with a threshold at 0.5 (Supplementary Table 2). One patient had serum GM negative test but proven IA (case 4), 2 patients had clinical and chest-CT scan patterns highly suggestive of IA (case 1 and 3). Finally, the fourth patient (case 2) had a clinical evolution less in favor of IA, despite the isolation of an *Aspergillus flavus* in sputum sample and a serum GM-ODI at 0.43. Indeed, GM test specificity decreases from 96.4% with an ICV at 0.5 to 85.9% with an ICV at 0.3.

## DISCUSSION

Despite antifungal prophylaxis, IA remains a diagnostic and therapeutic challenge in AML patients [4–6]. In a randomized multicentric study, Cornely *et al.* demonstrated that posaconazole reduces incidence of IFD and prolongs overall survival of patients with prolonged neutropenia [7]. This study leads to Food and Drug Administration approval of posaconazole for the prophylaxis of IFD in patients who are at high risk of developing these infections. The decrease of serum GM test sensitivity by antifungal prophylaxis has been demonstrated by Marr *et al*. in 2005 in patients that received chemotherapy or allogenic hematopoietic stem cell transplant for various hematological malignancies, but posaconazole was not used in this work [8]. So, the current observational and retrospective study was focused on a homogeneous population of AML patients receiving induction chemotherapy with posaconazole prophylaxis, in order to analyze breakthrough IA and diagnostic accuracy of GM assay.

Incidence of IA in this studied population was 8%, with 3.8% of probable and proven IA. This incidence is higher than the one described in prospective randomized study by Cornely *et al.* [7], but consistent with the incidence in real-life studies previously reported in AML patients [4, 9–12]. We found exclusively pulmonary IA with poor clinical symptoms. Fever was the sole symptom in 20% of IA patients. Those limited clinical manifestations could possibly be explained by antifungal prophylaxis. This point has already been described by Peterson *et al*. who reported IA-related clinical symptoms in 31% and 4%, without and with antifungal prophylaxis, respectively [13]. We found that prolonged neutropenia and low posaconazole plasma concentration were factors associated with high risk of IA [14, 15], but also chronic respiratory comorbidity. In such a context, patients with chronic respiratory disease could be screened for *Aspergillus* colonization before chemotherapy.

Antifungal prophylaxis is known to reduce the fungal load in animal models [16, 17] and serum GM level is known to be correlated to this fungal load [3], particularly in neutropenic patients: posaconazole could reduce serum GM load and might impair diagnostic accuracy of the test. In the paper from Marr *et al*., specificity of serum GM test remained unchanged but the authors showed a sensitivity for detection of probable and proven IA in control arm and treatment arm about 89% and 52%, respectively [8]. In the current study, all patients received antifungal prophylaxis by posaconazole oral suspension and sensitivity was 81.8% for a specificity about 96.4%. Moreover, we found that none patient with IA and posaconazole plasma concentration ≥ 0.5 mg/L had a serum GM positive test. We then tested a lower ICV and found an increased sensitivity of the serum GM test for probable and proven IA diagnosis. These results suggesting that posaconazole decreased the diagnostic accuracy of serum GM assay have been obtained in a specific population and should be taken with caution in other clinical situations. Indeed, the goal of an efficient prophylaxis is to lower the prevalence of a disease but the efficiency of a test is also highly dependent of this prevalence and spectrum of the disease [18, 19].

This is a critical point since posaconazole is now available in tablet formulation that has been developed to optimize bioavailability. Cornely *et al*. indeed recently showed that with this tablet formulation, the average concentration was superior to 0.5 mg/L for 99% of the patients [20]. Another study recently described posaconazole levels in real-life patients: the mean of posaconazole plasma concentration was 1.32 mg/L *versus* 0.81 mg/L, under tablet or oral suspension formulations, respectively [21].

Being aware of the drawbacks of our study design, we are convinced that our data make a valuable contribution in the field of IA risk management in AML patients during neutropenic period following induction chemotherapy. This study displays undoubtedly several bias due to its retrospective design and lack of systematic posaconazole plasma dosage (even if 90% had at least one dosage). We analyzed 2297 sera allowing strong multivariate analysis of risk factors associated with IA incidence. Whether these findings also translate into current practice with tablet formulation cannot be answered by our data but should be addressed by a prospective, multicentric study in the light of these results.

At our knowledge, posaconazole is not very prone to pharmacogenetic variability due to its low metabolism unlike voriconazole. If voriconazole is substrate and inhibitor of many isoforms of cytochrome P450, posaconazole is an inhibitor but only a weak substrate [22]. Actually, absorption is clearly the rate-limiting step for pharmacokinetics of posaconazole oral suspension. Other treatment interfere with results [23], particularly proton-pump inhibitors [24]. No data support interaction at distribution and elimination phases of pharmacokinetics and its metabolization is weak: the only drugs that could have an effect on these points are rifampicin and phenytoin that were not prescribed in these patients [25].

The last Infectious Diseases Society of America practice guidelines do not recommend anymore serum GM screening for asymptomatic patients receiving efficient mold-active antifungal prophylaxis [26]. The current study indeed suggests that AML patients with high posaconazole plasma concentration do not benefit from this screening policy. Such levels are mostly achieved with posaconazole tablet formulation, the galenic formulation currently used. Nevertheless, invasive aspergillosis remains a life-threatening infection that needs an early diagnosis. In our opinion, the IDSA recommendations should be taken with caution, only for patients with posaconazole plasma concentration ≥ 0.5 mg/L appended to a rigorous diagnostic-driven approach. The feasibility of this diagnostic-driven strategy has recently been assessed in a prospective study in high-risk hematology patients. The incidence of invasive fungal disease, fungal-related death and all-cause mortality rates were similar in a classical approach and in a diagnostic-driven strategy. However, only hundred patients were included in the diagnostic-driven strategy arm, oral solution and not tablet formulation of posaconazole was used, and no data on posaconazole plasma concentration were submitted [27]. Finally, other diagnostic tools could support new screening strategies. Even if A*spergillus* polymerase chain reaction is diminished under antifungal mold prophylaxis [28], its combination with serum GM assay have shown promising results [29, 30].

## MATERIALS AND METHODS

### Patients and prophylactic strategies

Selection criteria for this retrospective study were a diagnosis of AML according to WHO classification [31] between May 1st, 2008 and November 30, 2015 in the Department of Hematology and Cell Therapy of the Bordeaux University Hospital, > 17 years of age, without previous history of allogeneic or autologous hematopoietic stem cell transplant. Cytogenetic risk classification was defined according to the British Medical Research Council classification [32]. Patients have been included in the current study if they received at least 5 days of posaconazole prophylaxis (200 mg three times a day), exclusively by oral suspension. Posaconazole plasma concentration was monitored biweekly, started after 5 days of treatment [33]. If concentration was lower than 0.7 mg/L, posaconazole was increased to 200 mg four times a day. Written informed consents have been obtained from all patients in accordance with the Declaration of Helsinki, allowing the collection of clinical and biological data in an anonymized database, registered at the Commission Nationale Informatique et Libertés under N°1777604.

### Laboratory diagnosis of IFD

Diagnostic procedures for the diagnosis of IFD routinely used galactomannan enzyme immunoassay in serum, bronchoalveolar lavage (BAL), and cerebrospinal fluid (CSF) besides histology, direct examination and culture on clinical specimens according to standard method [34]. Briefly, identification of fungi was done using classical phenotypically methods until implementation of matrix-assisted laser desorption/ionization time-of-flight mass spectrometry (MALDI-TOF MS, Microflex, Bruker Daltonics): yeasts were identified using MALDI Biotyper V1.3 Software (Bruker Daltonics) since April 2013 and filamentous fungi were identified at the species level using an extended reference spectra library [35] since October 2014. Mucormycosis diagnosis was carried out using PCR on serum in the Mycology Laboratory of Besancon University Hospital [36]. Serum GM tests were performed twice a week until neutropenia recovery, yielding a total of 2297 collected sera. Galactomannan detection was performed using the Platelia™ *Aspergillus* Ag kit (Bio-Rad Laboratories, Marnes-la-Coquette, France) according to the manufacturer's instructions. Serum, BAL and CSF were treated under a class 2 biological safety cabinet. Positive GM tests were controlled on the same sample and always verified on another sample. GM assay in BAL were performed in duplicate.

### Monitoring of posaconazole plasma concentration

From December 1st, 2008 to February 15, 2010, the posaconazole plasma concentration was performed by high-performance liquid chromatography (limit of detection/LOD = 0.02 μg/mL and limit of quantification/LOQ = 0.05 μg/ml) assay developed for a simultaneous determination of systemic azoles (fluconazole, posaconazole, voriconazole, itraconazole and its metabolite hydroxyl-itraconazole, and ketoconazole) as previously published [37]. Since February 15, 2010, this posaconazole plasma concentration analysis is coupled with tandem mass spectrometry.

### Diagnostic strategy and EORTC/MSG 2008 sub group assignment

AML patient's characteristics are collected in an ongoing prospective database of the Department of Clinical Hematology and Cell Therapy. The clinical records of patients are evaluated to determine presence or absence of IA, date of diagnosis, date of clinical onset and receipt of antifungal therapies. Date of diagnosis was the earliest date among the day of suggestive computerized tomography (CT) scan or the day of microbiological or histopathological allowing diagnosis for probable and proven IA. The diagnosis of IFD was made in accordance with the revised EORTC/MSG definitions published in 2008 [2]. According to the above definitions, the whole cohort has host factor criteria with recent history of neutropenia (< 0.5×10^9^ neutrophils/L for > 10 days). Diagnosis of possible IA was made according to clinical criteria of lower respiratory tract fungal disease on CT-scan, trachea-bronchitis on bronchoscopic analysis, without mycological criteria. Diagnosis of probable IA was made from the documentation of one of the clinical or radiological findings described above associated with mycological criteria by isolation of mold in sputum, BAL or bronchial brush, or serum GM-ODI ICV > 0.5 or BAL GM-ODI ICV > 1. Diagnosis of proven IFD was retain by demonstrating fungus in diseased tissue (lobectomy or bronchial biopsy). All chest-CT scans have been routinely analyzed by a specialized radiologist (VL) and specifically reviewed for the study by an experienced pulmonary physician (EB) who was unaware of both clinical and biological results. CT images have been specifically examined in each lung and following radiological end points have been collected: nodule with or without halo sign, cavity / air crescent sign, ground glass opacities, alveolar consolidation, centrilobular micronodules, bronchiectases, pleural effusion, calcifications, and pneumothorax. Classical EORTC/MSG 2008 definitions were used for descriptive analysis of breakthrough IFD.

### Statistical analysis

Diagnostic accuracy of serum GM-ODI was estimated by computing and presenting as sensitivity and specificity under the conditions of the serum GM-ODI ICV of 0.3, 0.4 and 0.5 with their 95% confidence interval (CI). We used standard methods for binomial proportions to calculate the CI. The receiver operating characteristic (ROC) curve was calculated and the area under the curves was reported. We estimated association between explanatory variables and IA (binary dependent variable) by performing a multivariable logistic regression. Variables and interaction terms associated (p < 0.20) with IA status were then included in a multivariate logistic regression model (p < 0.20). The analyses were performed using SAS 9.3 (SAS institute Inc, Cary, NC) and R 3.2.2 [38].

## SUPPLEMENTARY MATERIALS FIGURES AND TABLES



## Abbreviations

AML: Acute Myeloid Leukemia
BAL: Broncho-Alveolar Lavage
CT: Computerized tomography
EORTC/MSG: European Organization for Research and Treatment of Cancer/Mycoses Study Group
GM: Galactomannan
GM-ODI: Galactomannan Optical Density Index
IA: Invasive Aspergillosis
ICV: Index Cutoff Value
IFD: Invasive Fungal Disease
IQR: Interquartile range
OR: Odds ratio
