# Supplementary material for: Breakthrough invasive aspergillosis and diagnostic accuracy of serum galactomannan enzyme immune assay during acute myeloid leukemia induction chemotherapy with posaconazole prophylaxis
Source: Oncotarget. 2018 Jun 1;9(42):26724–36. doi: 10.18632/oncotarget.25477 (PMC6003556; doi:10.18632/oncotarget.25477)
Supplement: Supplementary file 1 [file oncotarget-09-26724-s001.pdf]

Breakthrough invasive aspergillosis and diagnostic accuracy of serum galactomannan enzyme immune assay during acute myeloid leukemia induction chemotherapy with posaconazole prophylaxis

SUPPLEMENTARY MATERIALS

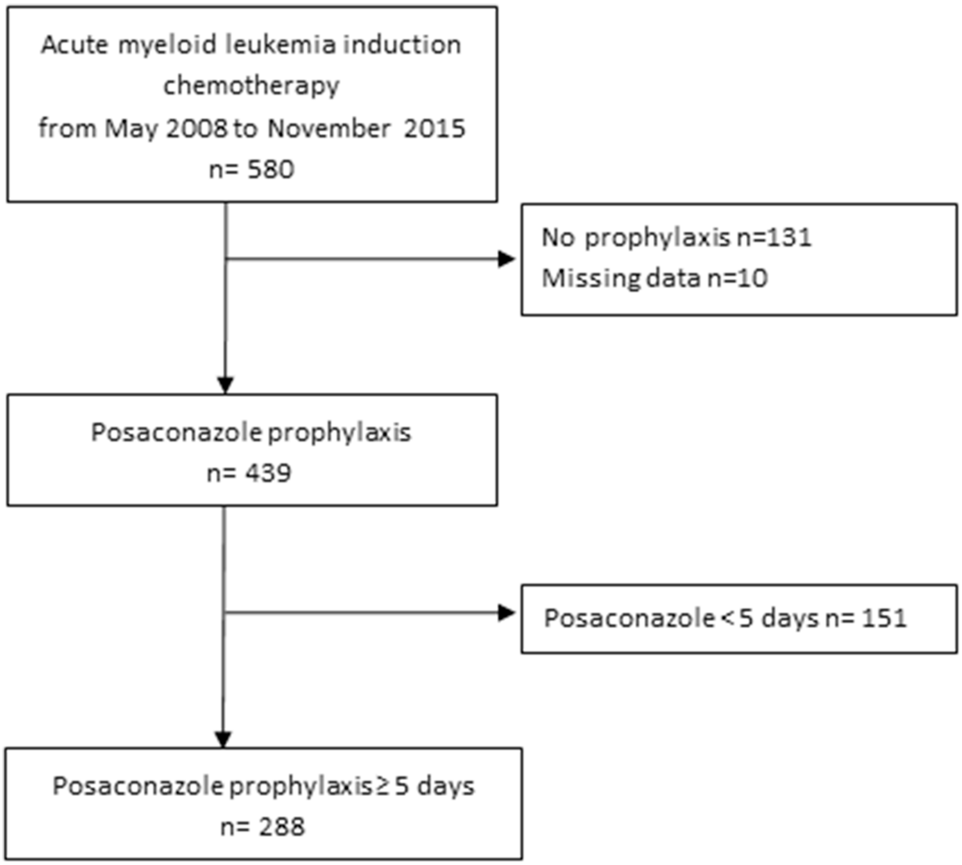

Supplementary Figure 1: Study flow-chart.

Supplementary Table 1: Schema of induction and reinduction chemotherapy

First induction course
- Cytarabine 200 $mg/m^2/d$ for 7 days + Daunorubicin 60 $mg/m^2/d$ for 3 days
- Cytarabine 100 $mg/m^2/d$ for 7 days + Idarubicin 8 $mg/m^2/d$ for 5 days
- Cytarabine 100 $mg/m^2/d$ for 7 days + Idarubicin 8 $mg/m^2/d$ for 5 days + Lomustin 200 mg/m^2 at day 1
- Cytarabine 500 $mg/m^2/d$ for 3 days + Daunorubicin 60 $mg/m^2/d$ for 3 days<br>Cytarabine 1000 $mg/m^2/12h$ on days 8 to 10 + Daunorubicin 35 $mg/m^2/d$ on days 8 and 9
- Cytarabine 200 $mg/m^2/d$ for 7 days + experimental treatment
**Second induction course**
- Cytarabine ≥ 1000 $mg/m^2/12h$ for 3 days

Supplementary Table 2: Four invasive aspergillosis detected by decreasing serum GM-ODI ICV from 0.5 to 0.3

	Case 1	Case 2	Case 3	Case 4
**Clinical signs**	Fever, dyspnea, crackles,	Fever, dyspnea	Fever, dyspnea, crackles,	Fever, cough
**CT signs**	Nodule with halo sign, pleural effusion, ground glass opacities	Condensation, micronodules, pleural effusion	Nodules with halo sign	Nodule with halo sign, ground glass opacities
**Colonization**	0	*Aspergillus flavus*[a]	0	0
**Serum GM-ODI**	0.46	0.43	0.34	0.37
**Bronchoscopy**	Inflammation	ND	Normal	Tracheobronchitis eschar
**Culture in BAL**	*Sterile mycelium*	0	0	*Aspergillus fumigatus*
**GM-ODI in BAL**	0.11	ND	0.12	ND
**Bacteriological documentation**	0	*Enterococcus faecium* & *Escherichia Coli* in blood	0	0
**Histopathology**	ND	ND	ND	1 positive bronchial biopsy
**EORTC/MSG**	Possible IA	Possible IA	Possible IA	Proven IA
**Evolution**	Clinical response Voriconazole discontinued after 12 weeks	Clinical response Voriconazole discontinued after 10 days	Clinical response Voriconazole discontinued after 7 months	Death from IA

CT: computerized tomography, BAL: bronchoalveolar lavage fluid, GM-ODI: Galactomannan optical density index, ND: not done, IA: invasive aspergillosis, EORTC/MSG: European Organization for Research and Treatment of Cancer/Mycoses Study Group.

[a] in a sputum performed two weeks before IA diagnosis, before the beginning of chemotherapy.
